# Total Lesion Glycolysis (TLG) on 18F-FDG PET/CT as a Potential Predictor of Pathological Complete Response in Locally Advanced Rectal Cancer After Total Neoadjuvant Therapy: A Retrospective Study

**DOI:** 10.3390/diagnostics15141800

**Published:** 2025-07-16

**Authors:** Handan Tokmak, Nurhan Demir, Hazal Cansu Çulpan

**Affiliations:** 1 Department of Nuclear Medicine, University of Health Sciences, Prof. Dr. Cemil Tascioglu Hospital, 34384 Şişli, Türkiye; 2 Istanbul Haseki Training and Research Hospital, 34096 Fatih, Türkiye; 3 Department of Public Health, Cerrahpasa Faculty of Medicine, Istanbul University-Cerrahpasa, 34098 Fatih, Türkiye; hazalcansu.acar@iuc.edu.tr

**Keywords:** rectal cancer, total neoadjuvant therapy, 18F-FDG PET-CT, total lesion glycolysis, pathological complete response

## Abstract

**Background:** The accurate prediction of pathological complete response (pCR) following total neoadjuvant therapy (TNT) is crucial for optimising treatment protocols in locally advanced rectal cancer (LARC). Although conventional imaging techniques such as MRI show limitations in assessing treatment response, metabolic imaging utilising 18F-fluorodeoxyglucose positron emission tomography/computed tomography (18F-FDG PET-CT) provides distinctive information by quantifying tumour glycolytic activity. This study investigates the predictive value of sequential 18F-FDG PET-CT parameters, focusing on Total Lesion Glycolysis (TLG), in predicting pCR after TNT. **Methods:** We conducted a retrospective analysis of 33 LARC patients (T3–4/N0–1) treated with TNT (neoadjuvant-chemoradiation followed by consolidation FOLFOX chemotherapy). Sequential PET-CT scans were performed at baseline, interim (after 4 cycles of FOLFOX), and post-TNT. Metabolic parameters, including maximum standardised uptake value (SUVmax) and TLG, were measured. Receiver operating characteristic (ROC) analysis assessed the predictive performance of these parameters for pCR. **Results:** The pCR rate was 21.2% (7/33). Post-TNT TLG ≤ 10 demonstrated excellent predictive accuracy for pCR (AUC 0.887, 92.3% sensitivity, 85.7% specificity, and 96.0% PPV), outperforming SUVmax (AUC 0.843). Interim TLG ≤ 10 also showed a strong predictive value (AUC 0.824, 100% sensitivity, and 71.4% specificity). **Conclusions:** TLG may serve as a reliable metabolic biomarker for predicting pathologic complete response (pCR) after total neoadjuvant therapy (TNT) in locally advanced rectal cancer (LARC). Its inclusion in clinical decision-making could improve patient selection for organ preservation strategies, thereby reducing the need for unnecessary surgeries in the future. However, given that the study is based on a small retrospective design, the findings should be interpreted with caution and used alongside other decision-making tools until more comprehensive data are collected from larger studies.

## 1. Introduction

### 1.1. Epidemiology and Clinical Significance of LARC

Colorectal cancer (CRC) ranks as the third leading cancer worldwide, with approximately 1.9 million new cases each year [1]. Locally advanced rectal cancer (LARC) characterised as T3–4 or node-positive (N+) disease without distant metastases, constitutes approximately 30% of all rectal cancer cases [2]. The conventional standard of care for LARC includes neoadjuvant chemoradiotherapy (CRT), followed by complete mesorectal excision (TME) and subsequent adjuvant chemotherapy [3,4,5]. These complications profoundly diminish patients’ quality of life, highlighting the need for more refined response-adaptive strategies that can spare patients unnecessary surgery when possible.

### 1.2. The Shift Toward Organ Preservation

The observation that 15–27% of LARC patients achieve a pathological complete response (pCR) after neoadjuvant therapy has spurred interest in non-operative “watch-and-wait” (W and W) strategies [6,7,8]. The International Watch and Wait Database (IWWD) reports five-year organ preservation rates of 85% in patients with a clinical complete response (cCR), with comparable survival outcomes to surgically treated patients [9]. Standard histopathology examination is the golden formula for evaluating the tumour response to preoperative radiochemotherapy (RCT). However, this approach is limited to the postoperative context and cannot be applied to the preoperative selection of customised therapy. Thus, the primary challenge to address is acquiring highly reliable data to accurately define the correct and complete answer without the need for surgery, while managing the patient within this framework. Current clinical assessments—including digital rectal examination (DRE), endoscopy, and MRI—have limited predictive performance, with false-negative rates of 10–20% [10,11,12]. This uncertainty underscores the need for more reliable biomarkers to distinguish true responders from those with residual disease.

### 1.3. Limitations of Conventional Imaging in Response Assessment

Magnetic resonance imaging (MRI), particularly diffusion-weighted imaging (DWI), is the cornerstone of post-treatment evaluation. However, it suffers from the following key limitations: (1) the inability to differentiate fibrosis from residual tumour and post-CRT fibrosis can mimic residual disease on T2-weighted MRI, leading to false positives [13]. (2) Moderate negative predictive value (NPV): studies report NPVs of 65–75% for pCR, meaning that 25–35% of patients deemed “complete responders” may harbour residual disease [14,15]. (3) Interobserver variability: MRI assessments are highly dependent on radiologist expertise, with kappa values as low as 0.4–0.6 for tumour regression grading (TRG) [16,17,18].

While endoscopic evaluation complements MRI, it cannot detect microscopic residual disease and has a false-negative rate of 15–20% in post-CRT settings [19]. These limitations highlight the urgent need for functional imaging modalities that can provide more data.

### 1.4. The Role of Metabolic Imaging in LARC

The process of 18F-FDG PET-CT offers a functional assessment of tumour metabolism, providing information independent of morphological changes for evaluating treatment response. The maximum standardised uptake value (SUVmax) has been widely studied. Still, it has limitations due to its reliance on a single voxel of maximal activity, which may not represent the entire tumour’s metabolic behaviour. In contrast, total lesion glycolysis (TLG), which incorporates both metabolic activity and tumour volume, may better reflect heterogeneous treatment responses [20,21,22].

### 1.5. PET-CT in Early Identification of Poor Responders

While much attention has focused on pCR prediction, PET-CT also plays a critical role in identifying poor responders—those with minimal metabolic response or progression during therapy. These patients are at a higher risk of distant metastases and poor long-term survival, necessitating early treatment intensification. Metabolic non-response on FDG-PET/CT is strongly associated with inferior survival in a study reporting that patients with <60% reduction in TLG post-CRT had a 5-year DFS of 52%, compared to 78% in good responders (*p* < 0.001) [15]. Sun et al. Demonstrated that patients with <30% reduction in TLG after CRT had a three-year distant metastasis rate of 45%, compared to 12% in good responders [23]. Janssen et al. found that early interim PET-CT (two weeks into CRT) could identify non-responders with 88% specificity, enabling early therapy modification [24]. Memon et al.’s review of FDG-PET Prediction of Complete Pathological Response and Survival in Rectal Cancer highlights that post-treatment SUVmax and response index for SUVmax appear to be useful FDG-PET markers for prediction of pCR, and these parameters also show strong associations with DFS and OS [25]. These findings underscore PET-CT’s dual role: selecting complete responders for organ preservation and identifying the poor responders who may benefit from early treatment escalation (e.g., chemotherapy intensification or metastasectomy).

### 1.6. The Scientific Rationale for Delayed PET-CT in pCR Assessment

A major confounder in post-CRT PET imaging is radiation-induced inflammation, which can mimic residual tumour activity. Studies show that inflammatory FDG uptake peaks at 2–4 weeks post-radiotherapy and gradually declines thereafter [24]. Early PET-CT (≤6 weeks post-CRT) has poor specificity (50–60%) due to false-positive uptake [25]. Our study addresses this by delaying PET-CT until 20 weeks post-radiotherapy and four weeks post-TNT completion, allowing inflammatory changes to resolve.

### 1.7. Study Rationale and Objectives

Despite advances in TNT, no consensus exists on the optimal PET-CT timing or parameters for pCR prediction. Our study aims to compare TLG and SUVmax at sequential timepoints (baseline, interim, and post-TNT) to identify the most accurate predictor of pCR. We also aimed to establish metabolic thresholds (e.g., TLG ≤ 10) to guide clinical decision-making for organ preservation and evaluate the prognostic value of early metabolic changes in identifying poor responders who may require treatment adaptation.

### 1.8. Study Design and Patient Population

This retrospective analysis included 33 LARC patients (T3–4/N0–1) treated with TNT (CRT + consolidation chemotherapy) between 2014 and 2019. Key exclusion criteria were metastatic disease and prior pelvic radiotherapy.

**Figure 1:**
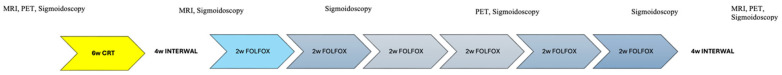
Imaging Protocol.

A complete colonoscopy was performed for histopathologic diagnosis and to obtain data on tumour location. Whole-body-staging was performed using PET/CT. In all patients, the pelvic MRI performed the locoregional staging with a special rectal cancer imaging protocol. All patients underwent baseline PET-CT, interim PET-CT during consolidation chemotherapy, final PET-CT 4 weeks post-TNT completion (Figure 1), interval post-TNT PET-CT, and surgery between 4 and 6 weeks. The median interval was 5.2 weeks (range: 4–6).

### 1.9. Innovation and Clinical Impact

Our study provides new insights by evaluating Total Lesion Glycolysis (TLG) in the context of modern TNT regimens, which are now standard but have been understudied in the PET literature. This research addresses this gap, allowing for more accurate PET-based response stratification. By implementing a 20-week interval after radiotherapy, we can reduce inflammatory artefacts caused by FDG uptake. This enhancement improves the reliability of predicting pathological complete response (pCR). Additionally, identifying practical thresholds for predicting pCR using TLG may establish clinically feasible parameters for pCR prediction, which could refine patient selection for Watch-and-Wait (W&W) strategies.

### 1.10. Treatment Protocol

TNT was applied to all patients (Figure 1). All patients were treated with image-guided intensity-modulated radiotherapy or volumetric intensity-modulated arc treatment using 6–10 MV photons. Patients received a pelvic radiotherapy dose of 50.4/Gy delivered in 28 fractions and concomitant oral capecitabine 825 mg/m^2^ twice daily during radiotherapy. After four weeks, all patients were reevaluated by sigmoidoscopy and pelvic MRI. Six cycles of consolidation chemotherapy were administered, comprising bi-weekly FOLFOX administration (oxaliplatin-[85 mg/m^2^] and concomitant leucovorin-[400 mg/m^2^] for two hours followed by a bolus injection of 5-fluorouracil [400 mg/m^2^]. Then, 5-fluorouracil-[2400 mg/m^2^] was infused over 46 h) after CRT. Regarding the tolerability of TNT, all patients completed the chemotherapy course. In terms of negative consequences, the majority were categorized as grade 1 or 2. All response evaluations were performed during the study using endoscopy, MRI, and PET/CT (Figure 1).

### 1.11. PET-CT Acquisition and Analysis

Pretreatment, interim (after four cycles of the Folfox regimen), and post-TNT (completed six cycles of Folfox and a four-week interval period) PET-CT scans were obtained, and all patients underwent surgery. Post-consolidation PET-CT images were reviewed for FDG uptake in the primary tumour. PET-CT parameters were analysed for metabolic response at the primary tumour area and compared across different stages.

**Figure 2:**
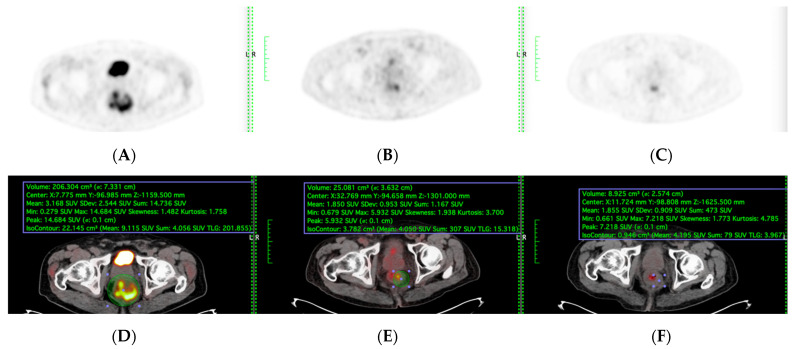
(**A**,**D**) Before, (**B**,**E**) during, and (**C**,**F**) after TNT PET and PET-CT fusion images were included in TLG quantification. Scanner: Siemens Biograph LSO HI-REZ True-X HD PET-CT.

PET-CT, including maximum standardised uptake value (SUVmax) and total lesion glycolysis (TLG), were evaluated among the patients who underwent sequential PET-CT before, during, and after TNT were included (Figure 2). Total Lesion Glycolysis (TLG) was automatically calculated using vendor-specific software by multiplying the metabolic tumour volume (MTV) by the mean standardised uptake value (SUVmean) within the volume of interest (VOI). Lesion boundaries were delineated using a fixed SUV threshold of 2.5 with manual adjustment to exclude physiologic bowel uptake and adjacent organs, consistent with PERCIST guidelines. Sensitivity, specificity, accuracy, and areas under the model’s receiver operating characteristic curve (AUC) were evaluated to determine its performance.

**Figure 3:**
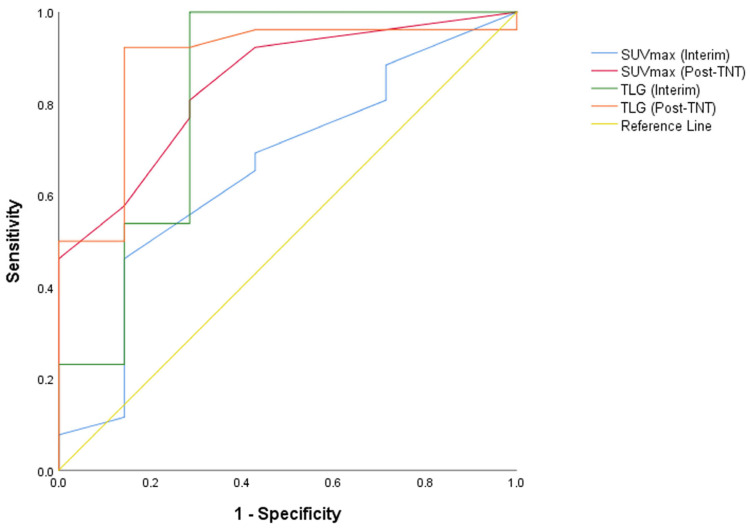
Receiver Operating Characteristic (ROC) Curves for Interim and Post-TNT PET/CT Parameters. For predicting treatment response, post-TNT TLG (red curve) exhibits the best trade-off between sensitivity and specificity. The blue curve, or interim SUVmax, may provide early but imprecise response signals.

All patients fasted for at least 6 h before the PET/CT scan. Prescanning blood glucose was systematically checked and adjusted to less than 150 mg/dL. Patients received an intravenous injection of 18F-FDG, at a dose of 3.7 MBq/kg of body weight. After 60 min of uptake, the patient was positioned on a flat tabletop, using a movable laser-alignment system, in a “head-first supine” position. A PET-CT scanning of the whole body (six or seven bed positions from head to thigh) was performed using an acquisition time of 2 min per bed position. For each of the PET images, a tumour contour was generated using automated SUVs, which reached threshold levels (SUV > 2.5). The metabolic parameters were acquired and calculated as follows: SUVmax and total lesion glycolysis (TLG). The response is the percentage difference between the results of the baseline PET-CT scan and the results of the other two PET-CT scans) for SUVmax and TLG. SUVmax and TLG were measured at the primary tumour spot (Figure 3).

#### Pathological Assessment

Surgical resection was performed 8–10 weeks after completion of TNT. Pathological complete response (pCR) was defined as ypT0N0 (no viable tumour cells in the primary tumour or lymph nodes). Non-pCR cases were subclassified as ypT1–3.

### 1.12. Statistical Analysis

Statistical analyses were performed using SPSS v26.0 (IBM Corp., Armonk, NY, USA). The ShapiroWilk Test was used to evaluate the normality. Continuous variables were presented as mean ± standard deviation and range (minimum–maximum) and analysed with Student’s *t*-test. Categorical variables were presented as frequency and percentage and analysed with Fisher’s Exact test. The changes in SUVmax and TLG were assessed by using the Friedman test. The Receiver Operating Characteristic (ROC) curve was used to evaluate the predictive ability of TLG on pathological complete response in locally advanced rectal cancer after total neoadjuvant therapy. The predictive ability is assessed by using the area under the curve (AUC), sensitivity, specificity, positive predictive value (PPV), and negative predictive value (NPV). A *p*-value < 0.05 was accepted for statistical significance.

## 2. Results

### 2.1. Patient Characteristics

A total of 33 patients with a mean age of 54.9 ± 12.5 (range: 28–73) were included in study (Table 1). Of the 33 patients, 57.6% (*n* = 19) were male. The percentage of pCR was 21.2% (*n* = 7) while the percentage of non-pCR was 78.8% of which 42.4% (*n* = 14) was ypT2 and 36.4% (*n* = 12) was ypT3. There was no significant difference between pCR and non-pCR in terms of age, sex, tumour location, histology, and ypN+.

**Figure 4:**
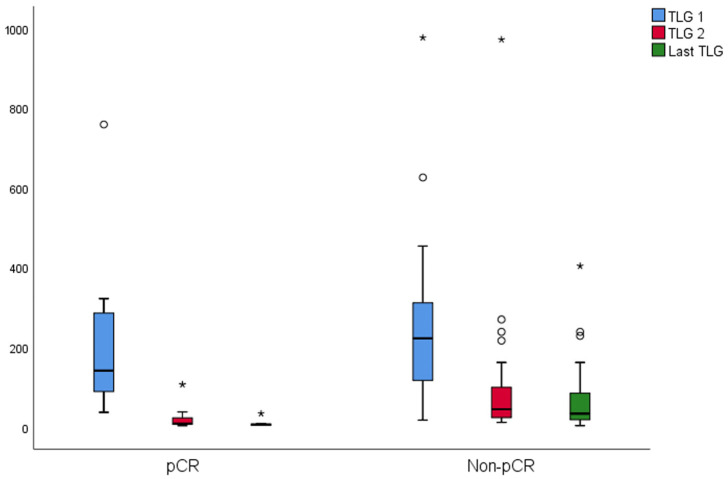
Box plot displaying SUVmax changes at three time points: Baseline (SUVmax 1), interim (SUVmax 2), and post-treatment (Last SUVmax). A circle means outlier (3rd quartile + 1.5 × interquartile range) whereas an asterisk means extreme outlier (3rd quartile + 3 × interquartile range).

**Figure 5:**
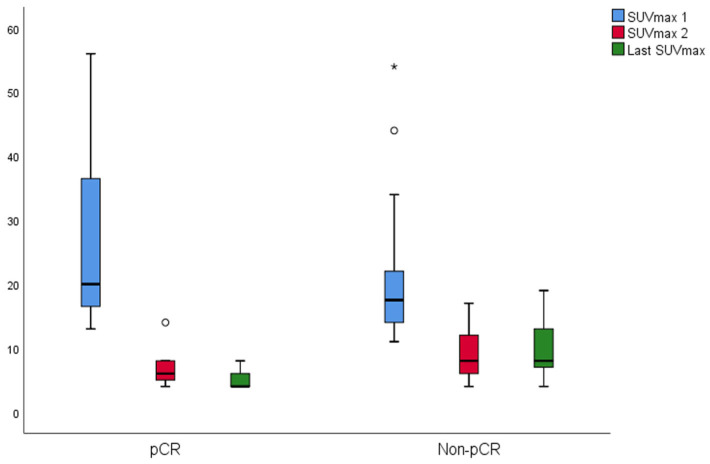
SUVmax dynamics are presented in a box plot at three timepoints: baseline (SUVmax 1), interim (SUVmax 2), and post-treatment (last SUVmax). A circle means outlier (3rd quartile + 1.5 × interquartile range) whereas an asterisk means extreme outlier (3rd quartile + 3 × interquartile range).

The changes in SUVmax and TLG of the groups are shown in Figure 4 and Figure 5, respectively. In the pCR, there was a significant difference between SUVmax values (*p* = 0.001). The last SUVmax was significantly lower than the SUVmax 1 (*p* = 0.003). The median SUVmax 2 was lower than SUVmax 1; however, it was not significant (*p* = 0.069). In the non-pCR, there was a significant difference between SUVmax values (*p* < 0.001). Both last SUVmax and SUVmax 2 were significantly lower than the SUVmax 1 (*p* < 0.001 both). In the pCR, there was a significant difference between TLG values (*p* = 0.004). Both last TLG and TLG 2 were significantly lower than the TLG 1 (*p* = 0.006, *p* = 0.033 respectively). In the non-pCR, there was a significant difference between TLG values (*p* < 0.001). Both last TLG and TLG 2 were significantly lower than the TLG 1 (*p* < 0.001 both).

### 2.2. Predictive Performance of PET-CT Parameters

Different cutoff values in the same PET-CT metabolic quantitative data, such as SUVmax and TLG at the second (interim) and the PET-CT after completed TNT (post-TNT), were noted to determine the most accurate metabolic response evaluation period and measurement method to predict pCR.

Retrospective analysis revealed that preoperative PET-CT (after completing TNT) TLG with a cutoff of 10 demonstrated significant predictive value (AUC: 0.887; *p* = 0.002), with a high positive predictive value (96.0%), sensitivity (92.3%), and specificity (85.7%). Interim PET-CT TLG with a cutoff of 10 also showed a significant predictive value (AUC: 0.824; *p* = 0.009), with high sensitivity (100%), a negative predictive value (100%), and a positive predictive value (92.9%). Preoperative SUV-max with a cutoff of 5 showed a significant predictive value (AUC: 0.843; *p* = 0.006), with a moderately high positive predictive value (91.3%), sensitivity (80.8%), and specificity (71.4%). However, interim SUV-max with a cutoff of 5 had 0.657 AUC and *p* = 0.209. PET-CT TLG (Post-TNT) was statistically superior to preoperative SUV-max (*p* = 0.007), but no statistical difference was found among others (*p* < 0.05). ROC curves of PET-CT parameters are given in Figure 4, and the predictive accuracy of PET-CT parameters for pCR are given in Table 2.

## 3. Discussion

The ability to accurately predict pathological complete response (pCR) in locally advanced rectal cancer (LARC) after total neoadjuvant therapy (TNT) is critical for guiding organ preservation strategies. Our study demonstrates that metabolic imaging parameters—particularly Total Lesion Glycolysis (TLG)—measured at an extended interval after radiotherapy completion (20 weeks) and 4 weeks after TNT completion, improve pCR prediction compared to conventional early post-treatment assessments. This finding aligns with emerging evidence that prolonged waiting periods after chemoradiation allow for the resolution of inflammatory changes, thereby enhancing the specificity of 18F-FDG PET-CT in distinguishing true complete responders from partial responders with residual inflammation.

### 3.1. The Challenge of Post-Treatment Inflammation in Early PET Imaging

A major limitation of using 18F-FDG PET-CT for response assessment shortly after radiotherapy is the confounding effect of radiation-induced inflammation, which can mimic residual tumour activity. Several studies have reported that inflammatory cells (e.g., macrophages and fibroblasts) exhibit increased glucose metabolism, leading to false-positive interpretations when imaging is performed too soon after therapy [26].

For example, Capirci et al. observed that PET-CT performed within 2–4 weeks after chemoradiotherapy (CRT) had poor specificity (50–60%) due to persistent inflammatory uptake, despite moderate sensitivity (70–80%) for detecting residual disease [27]. Similarly, Janssen et al. found that delayed imaging (>8 weeks) improved accuracy [28,29].

Our study’s delayed imaging protocol (20 weeks post-radiotherapy, 4 weeks post-TNT) addresses this limitation by allowing sufficient time for inflammatory changes to subside. This approach is supported by preclinical data showing that radiation-induced inflammation peaks at 2–4 weeks post-treatment and gradually declines thereafter [30,31,32]. In line with our findings, a recent study by in the OPRA trial demonstrated that a longer interval between neoadjuvant therapy and surgery improved the correlation between clinical complete response (cCR) and pCR, suggesting that delayed assessments reduce false positives [33].

### 3.2. TLG as a Superior Biomarker: Biological and Technical Rationale

Although SUVmax remains the most commonly reported PET parameter, our data indicate that TLG outperforms SUVmax in pCR prediction (AUC 0.887 vs. 0.843). However, the comparison between post-TNT SUVmax AUC and TLG AUC was not statistically significant. Both TLG and post-TNT SUVmax produced statistically significant results in predicting pCR. Additionally incorporating effective parameters like TLG, which combines metabolic activity and tumour volume, would improve clinicians’ confidence when making management decisions. While SUVmax remains the most widely reported PET parameter, our data indicate that TLG outperforms SUVmax in pCR prediction (AUC 0.887 vs. 0.843). This aligns with multiple studies across different cancers, reinforcing TLG’s role as a composite metric that integrates metabolic activity and tumour volume as shown below:

1. Intratumoural Heterogeneity: Unlike SUVmax (a single-pixel value), TLG accounts for spatial heterogeneity, which is crucial in LARC where residual tumour cells may be scattered rather than concentrated in a single focus [19,20].
2. Early Metabolic Changes: TLG reductions often precede morphological shrinkage on MRI, making it valuable for interim response assessment. A study by Sun et al. found that an SUVmax reduction by ≥50% post-CRT correlated with pathological response (*p* = 0.01) and further reported that TLG after CRT was more predictive of pCR than SUVmax [23].
3. Volume-Dependent Prognostication: TLG’s incorporation of metabolic tumour volume (MTV) may better reflect total disease burden.
4. Our TLG cutoff of ≤10 achieved a 96% PPV for pCR, suggesting that patients meeting this threshold could be strong candidates for non-operative management. This finding is particularly relevant given the growing interest in the “watch-and-wait” approach, where false-positive selections can lead to undertreatment.

### 3.3. Comparative Evidence from Other Cancers

The principle of delayed PET imaging for improved specificity is not unique to rectal cancer. In oesophageal cancer, where post-CRT inflammation similarly complicates early response assessment, studies have shown that PET-CT performed ≥12 weeks after CRT improves diagnostic accuracy. Ku et al. demonstrated that delayed PET-CT (12 weeks post-CRT) significantly outperformed early imaging (4 weeks) in predicting pCR (AUC 0.85 vs. 0.67, *p* = 0.003) in oesophageal cancer [34], mirroring our findings in rectal cancer where a 20-week post-radiotherapy PET-CT improved specificity by allowing inflammatory changes to resolve. The AUC improvement with delayed imaging in Ku’s study supports our protocol of extended intervals between therapy completion and metabolic assessment. This parallels findings in rectal cancer, whereby Van Stiphout reported AUC improvements from 0.68 to 0.86 when incorporating delayed PET data [35].

In lymphoma, the *Deauville criteria* recommend a 6–8 week post-treatment interval, and the Lugano Classification lymphoma response criteria recommend a 6–12 week post-treatment interval for PET-CT to avoid false positives from inflammatory changes [36,37,38]. This parallels our approach of extending the interval to 20 weeks post-radiotherapy, which optimises the balance between sensitivity and specificity.

### 3.4. Combining Functional Imaging and Biomarkers for Enhanced Accuracy

While TLG alone shows strong predictive value, multimodal integration may further refine pCR assessment.

- PET/MRI Hybrid Imaging: Simultaneous acquisition of metabolic (PET) and functional MRI data (DWI, perfusion) could improve spatial correlation. A pilot study by Grueneisen et al. in rectal cancer found that PET/MRI had higher accuracy (AUC 0.92) than PET or MRI alone [39].
- Blood-Based Biomarkers: Circulating tumour DNA (ctDNA) and inflammatory markers (e.g., IL-6) may complement imaging. Tie et al. demonstrated that ctDNA clearance after TNT predicted pCR with 98% specificity in LARC [40].
- Radiomics: Machine learning models incorporating PET texture features have shown promise [41]. A study by Yip et al. (2017) achieved an AUC of 0.94 by combining TLG with radiomic signatures [42].

### 3.5. Clinical Implications and Future Directions

Our findings support two key clinical strategies: Extended Waiting Periods: Delaying PET-CT until 20 weeks post-radiotherapy reduces inflammatory artifacts, improving pCR prediction. This could refine patient selection for organ preservation. Interim TLG for Adaptive Therapy: Early TLG reductions (e.g., mid-TNT) may identify patients who could benefit from intensified consolidation chemotherapy, as seen in the RAPIDO trial [43].

Novel PET tracers, such as fibroblast activation protein inhibitors (FAPI), are produced by cancer-associated fibroblasts (CAFs) within the tumour stroma. These tracers play a vital role in remodelling the extracellular matrix (ECM) by breaking down collagen and promoting tumour growth through processes like angiogenesis and immunosuppression. FAPI tracers, including [68Ga]Ga-FAPI-04, specifically bind to FAP and offer significantly improved tumour-to-background ratios compared to FDG in malignancies that are typically FDG-avid, such as pancreatic and gastric tumours. Moreover, their minimal physiological absorption in the intestines and brain enhances their utility for rectal imaging.

In addition to standard FDG-PET, fibroblast-targeted [68Ga]Ga-FAPI-04 PET shows promise for evaluating responses in rectal cancer. The low intestinal background and specificity of FAPI for CAFs, in contrast to FDG, may lead to better interpretation of post-chemoradiotherapy (CRT) outcomes, especially in mucinous subtypes.

For example, a 2023 pilot study reported that CRT predicted pathologic complete response (pCR) with an accuracy of 88%, while FAPI SUVmax decreased it by 60% or more. Although this finding aligns with our FDG-TLG data, it could offer more specific insights into stromal response. The process of selecting non-operative candidates may be further enhanced by combining FAPI with FDG or MRI.

In prospective studies, 18F-FDG PET/CT could serve two key roles in novel TNT regimens: early response assessment (e.g., interim SUVmax/TLG drop%) to identify non-responders for therapy escalation/de-escalation, and post-treatment metabolic complete response (mCR) definition to guide organ preservation. For example, mCR could complement MRI for Watch-and-Wait candidate selection, reducing unnecessary surgeries.

While our TLG cutoff (≤10) showed high PPV, it should complement—not replace—MRI and endoscopic assessments in multimodal algorithms (e.g., PET + MRI + ctDNA) to enhance decision-making.

The small sample size (*n* = 33) and the low number of pCR events (*n* = 7) limit the generalisability of our results. Although TLG ≤ 10 exhibited a high PPV (96%) for pCR in this cohort, larger prospective studies are essential to confirm its role in clinical decision-making.

### 3.6. Limitations of the Study

The retrospective design and small cohort (*n* = 33) limit generalisability. While TLG shows promise, prospective validation in larger studies is essential before clinical adoption.

## 4. Conclusions

**Figure 6:**
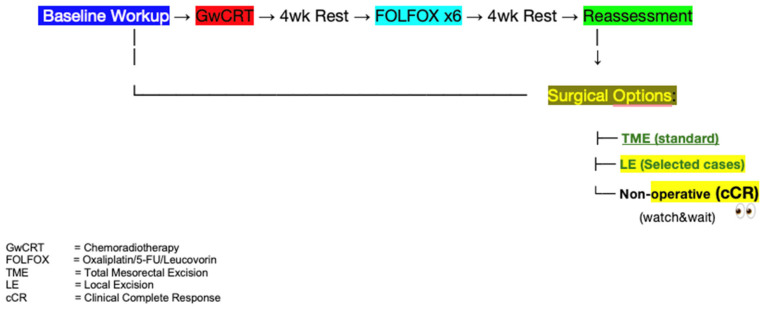
Patient management (TME standard). With reliable predictive methods, LE and watch-and-wait became options in selected patients.

The precise identification of pathologic complete response (pCR) is crucial for preventing unnecessary surgeries and the associated complications Figure 6. When conventional imaging techniques have limitations, 18F-FDG PET-CT offers a functional evaluation of tumour metabolism, providing valuable insights into treatment response. However, the retrospective nature and small sample size of this study limit the generalisability of our findings. Future research should aim to integrate functional imaging with molecular biomarkers to enhance predictive accuracy further and validate these results in larger, prospective cohorts. Furthermore, additional studies are necessary to confirm these findings and to explore the potential of combining metabolic imaging with other biomarkers. The total lesion glycolysis (TLG) measured on post-treatment 18F-FDG PET-CT is a strong predictor of pCR in locally advanced rectal cancer (LARC), surpassing SUVmax in effectiveness. Incorporating TLG into clinical decision-making could optimise organ preservation strategies.

In recent years, a comprehensive approach to predict pCR has emerged based on a combination of patient pathology, laboratory results, metabolic data, and radiological data findings, which has led to paradigm shifts in the management of LARC patients.

Currently, prospective studies that focus on FDG PET-CT as a crucial component of metabolic imaging are underway. Looking ahead, multi-center studies, enhanced radiological studies, deep learning programs, and radiomics that integrate clinical, radiological, and laboratory data will likely yield highly reproducible results.

Our study reinforces TLG as a robust predictor of pCR in LARC, particularly when assessed after an extended post-treatment interval. By mitigating the confounding effects of inflammation, this approach enhances the reliability of metabolic imaging for guiding non-operative management. As TNT becomes standard for LARC, integrating delayed PET-CT with emerging biomarkers and advanced imaging techniques will be essential for precision oncology.

Radiomics is computational imaging that involves using imaging data obtained from routine clinical evaluations to analyze tumor characteristics such as spatial heterogeneity, texture, and shape. This approach transforms imaging into a high-throughput data resource that can be analyzed alongside other clinical features to support precision medicine and enhance decision-making. Radiomics potential is currently being investigated in various clinical settings, including studies related to rectal cancer.

Key Recommendations:

1. Adopt TLG thresholds (≤10 post-TNT) in clinical trials focused on non-operative management of patients.
2. Validate findings in larger cohorts to establish standardised cutoffs.
3. Research TLG-guided adaptive therapy, including chemotherapy intensification for interim non-responders.
4. Use PET-CT’s functional insights to allow clinicians to go beyond anatomical imaging constraints and personalise treatment for LARC patients to maximise oncologic and functional results.

Future multi-center prospective studies with pre-planned statistical power calculations are needed to validate total lesion glycolysis (TLG) thresholds and to optimise patient selection for organ preservation.

## Figures and Tables

**Table 1:** Patients Characteristics.

	Total (*n* = 33)	pCR (ypT0, *n* = 7)	Non-pCR (ypT2-3, *n* = 26)	*p*-Value
Age (years)	54.9 ± 12.5	51 ± 14	55.4 ± 13.2	0.358
ECOG performance				
0	25 (75.76)	6		
1	8 (24.24)	1		
Male: Female	19:14	4:3	15:11	1.000
Tumour Location				1.000
Distal	28 (84.84%)	6 (85.7%)	22 (84.6%)	
Mid	4 (12.12%)	1 (14.3%)	3 (11.6%)	
Mid/Distal	1 (3.04%)	0 (0%)	1 (3.8%)	
Histology				1.000
Adenocarcinoma	31 (93.9%)	7 (100%)	24 (92.3%)	
Cribriform Comedo Adeno Ca.	2 (6.1%)	0 (0%)	2 (7.7%)	
Initial MR TNM stage				0.062
T3	20	4		
T4	13	3		
N+				
N−				
M	0	0		
Pathologic Stage				
T0	7	7		
T1	0			
T2	14			
T3	12			
N+	5 (15.1%)	0 (0%)	5 (19.2%)	0.559
N−	28			
Initial CEA				0.041
>5	12	1	11	
<5	21	6	15	
Post TNT CEA				0.545
>5	3	0	3	
<5	30	7	23	

**Table 2:** Predictive accuracy of PET-CT parameters for pCR.

Parameter	Time Point	Cutoff	AUC (95% CI)	*p*-Value	Sensitivity	Specificity	PPV	NPV
TLG	Post-TNT	≤10	0.887 (0.74–1.00)	0.002	92.3%	85.7%	96.0%	75.0%
SUVmax	Post-TNT	≤5	0.843 (0.69–1.00)	0.006	80.8%	71.4%	91.3%	50%
TLG	Interim	≤10	0.824 (0.60–1.00)	0.009	100%	71.4%	92.9%	100%
SUVmax	Interim	≤5	0.657 (0.42–0.89)	0.209	80.8%	28.6%	80.8%	28.6%

AUC: Area under curve, CI: confidence interval, PPV: positive predictive value, NPV: negative predictive value, TLG: total lesion glycolysis, SUVmax: maximum standardized uptake value, and TNT: total neoadjuvant therapy.

## Data Availability

The raw data supporting the conclusions of this article will be made available by the authors on request.
